# Complete Genome Sequences of Human Japanese Encephalitis Virus Genotype V Isolates in Korea Reveal Genotype-Specific Amino Acid Signatures

**DOI:** 10.3390/pathogens14121279

**Published:** 2025-12-12

**Authors:** Seung-Rye Cho, Ye-Ji Lee, Myung Guk Han, Heui Man Kim

**Affiliations:** Division of Viral Diseases, Department of Laboratory Diagnosis and Analysis, Korea Disease Control and Prevention Agency, 187 Osongsaengmyeong 2-ro, Osong-eup, Cheongju-si 28159, Republic of Korea; altodanf99@korea.kr (S.-R.C.); dana6532@korea.kr (Y.-J.L.); mghan@korea.kr (M.G.H.)

**Keywords:** Japanese encephalitis virus, genotype V, cerebrospinal fluid, amino-acid signature, dual clades, mosquito lineages

## Abstract

Japanese encephalitis virus (JEV) is a mosquito-borne zoonotic flavivirus causing severe neurological disease across Asia, and genotype V (GV) is now predominant in Korea. Despite frequent detection of GV in mosquitoes, human-derived complete genome data remain scarce. To elucidate the molecular and antigenic characteristics of human GV infections, cerebrospinal fluid samples from unvaccinated patients positive for JEV RNA during 2018–2023 were subjected to virus isolation in LLC-MK2 cells (rhesus monkey kidney-derived epithelial cell line). Three human GV isolates (K18P80, K23P84, K23P88) were successfully obtained and their complete open reading frames (~10.3 kb) sequenced. Phylogenetic analysis with representative JEV strains (GI–GV) revealed that these isolates form a distinct lineage, clustering into two domestic clades (Clade I and II), suggesting endemic circulation and local evolution in Korea. Sequence identities with GIII-based vaccine strains were low (79% nucleotide, 91.1% amino acid), with notable divergence in nonstructural regions. Three consistent E protein substitutions (Q52E, S156T, D292E) near antigenic epitopes indicate possible immune escape. Additional clade-defining substitutions in NS3 (L31F) and NS5 (K269R, M330I) were shared with mosquito isolates, supporting human–vector molecular continuity. These findings provide fundamental genomic evidence of human JEV GV in Korea and highlight the need for genotype-specific surveillance and next-generation vaccine evaluation.

## 1. Introduction

Japanese encephalitis virus (JEV) is a mosquito-borne member of the genus *Orthoflavivirus* belonging to the family *Flaviviridae* and is among the most prominent zoonotic arboviruses in Asia, causing considerable morbidity and mortality each year [1]. The World Health Organization (WHO) reports that the annual incidence of clinical JE disease varies across endemic countries and warns that estimates likely underestimate the true burden [2]. A systematic review estimated that approximately 100,000 JE cases and 25,000 deaths occur each year in regions where JEV circulates [3]. More than half of survivors experience permanent neurological sequelae, highlighting JE as a persistent public health and socioeconomic challenge [4]. JEV consists of a single-stranded, positive-sense RNA genome encoding three structural proteins (C, prM, and E) and seven nonstructural proteins (NS1, NS2A, NS2B, NS3, NS4A, NS4B, and NS5). Based on nucleotide sequence homology, JEV strains are classified into five genotypes: GI, GII, GIII, GIV, and GV [5,6]. Historically, GIII was the predominant genotype across Asia, but in recent decades, GI has emerged as the dominant genotype [7]. Additionally, GIV has recently emerged in Australia with a notable outbreak, highlighting the dynamic genotype shifts and their public health implications [8]. The Muar strain, first isolated in Malaysia in 1952, was later identified as belonging to genotype V (GV), which remained largely unreported for more than 50 years until its re-emergence in China in 2009 [9]. In Korea, GV has been continuously detected since the early 2010s through entomological surveillance, found in *Culex tritaeniorhynchus*, *Culex orientalis*, and *Culex pipiens* [10]. These observations suggest the potential for endemic circulation and an expanded range of competent vectors.

In the Republic of Korea, prior to the introduction of the JE vaccine in the early 1970s, thousands of cases and hundreds of deaths occurred annually. After the expansion of the national immunization program in the 1980s, there was a marked decrease in incidence; however, since 2010, annual case reports have persisted, ranging from 10 to 40 cases per year. From 2010 to 2023, a total of 275 cases and 40 deaths have been recorded. The peak incidence was in 2015, with 40 reported cases, coinciding with the first isolation of a human-derived GV strain (K15P38) from cerebrospinal fluid (CSF), providing direct evidence of GV infection in humans [11]. Currently available JE vaccines, both domestically and internationally, are derived from GIII strains. Recent studies have shown that GV has distinct genetic and antigenic profiles compared to GIII, resulting in reduced neutralizing activity and potentially lower vaccine efficacy [12,13]. Additionally, GV exhibits higher neurovirulence in vivo, with notable blood–brain barrier disruption, unique inflammatory responses, and histopathological changes unlike those caused by GIII [14]. Amino acid substitutions in major structural proteins, especially the E protein, are implicated as key factors driving antigenic variation and immune evasion [15,16,17]. Despite increasing detection of GV in mosquitoes, complete genome sequences from human GV isolates remain extremely scarce. Molecular characterization of human-derived GV and investigations into its genetic relationship with mosquito-borne strains are critical to understanding its circulation and pathogenic evolution. In the present study, we report three complete GV genome sequences obtained between 2018 and 2023 from CSF of JE patients in Korea. We analyzed the molecular genetic features of these human isolates and compared them with mosquito-derived strains, providing new insights into the ecology of GV circulation. These findings provide essential baseline data to guide JE vaccine efficacy assessment and inform improvements to national and regional surveillance programs.

## 2. Materials and Methods

### 2.1. Sample Collection and Diagnosis of Japanese Encephalitis

In the Republic of Korea, JE is designated as a class 3 notifiable infectious disease. All suspected cases are subject to nationwide sentinel surveillance, rapid diagnosis, and mandatory reporting. In this study, acute-phase clinical specimens (CSF and serum) were collected from patients suspected of JE based on clinical symptoms. Upon receipt at the laboratory, all specimens were immediately aliquoted and stored at −80 °C until testing. Storage duration varied by year of collection, ranging from several months to up to three years, and all samples were maintained at −80 °C without freeze–thaw cycles. Molecular diagnosis using real-time reverse transcription PCR (real-time RT-PCR) was combined with serological testing (JEV-specific IgM ELISA) to confirm infection. When necessary, convalescent-phase specimens were also collected to evaluate a ≥4-fold increase in antibody titers compared to the acute phase, confirming the final JE diagnosis.

Molecular detection targeted the NS5 region of the JEV genome, performed with the ABI 7500 Real-Time PCR System (Applied Biosystems, Foster City, CA, USA). Reactions used the GeneFinder™ JEV/TBEV RealAmp Kit (Osang Healthcare Co., Ltd., IFMR-58, Anyang-si, Gyeonggi-do, Republic of Korea). Serological assays employed the commercially available JE Detect™ IgM Capture ELISA for Japanese Encephalitis (InBios, JEMS-1, Seattle, WA, USA), following the manufacturer’s instructions.

### 2.2. Specimens Included in the Study

Clinical specimens were obtained during JE transmission seasons in September 2018 and September 2023 from patients who were hospitalized for laboratory confirmation of JE at sentinel hospitals in Korea. Among the specimens tested, three CSF samples from unvaccinated patients were positive for JEV RNA by real-time RT-PCR (*n* = 3), and only these were included for virus isolation and genomic analysis. All specimens used for virus isolation were stored at −80 °C from the time of collection until use, with no freeze–thaw cycles to preserve viral RNA integrity.

Virus isolation from these three CSF samples yielded one strain from 2018 (K18P80) and two from 2023 (K23P84 and K23P88). All isolates were subjected to complete genome sequencing and phylogenetic analysis.

This study was approved for exemption from review by the Korea Disease Control and Prevention Agency Institutional Review Board (IRB) (Approval No. KDCA-2025-08-06-PE-01).

### 2.3. Virus Isolation

For virus isolation, clarified CSF supernatant was inoculated onto LLC-MK2 cells, a rhesus monkey kidney–derived epithelial cell line (ATCC CCL-7, American Type Culture Collection, Manassas, VA, USA). Cells were maintained in Minimum Essential Medium (MEM; Gibco, Thermo Fisher Scientific, Waltham, MA, USA) supplemented with 10% fetal bovine serum (FBS; Gibco) and penicillin/streptomycin at 37 °C under 5% CO_2_.

Cultures were monitored daily until cytopathic effects (CPE) appeared. The CPE observed in infected LLC-MK2 cells included cell rounding, shrinkage, reduced adherence, and formation of small cell clusters, which typically appeared 3–5 days post-inoculation. Virus replication was confirmed by real-time RT-PCR using the same diagnostic reagents described above. Isolates were propagated with 1–2 passages to amplify viral stocks. Viral RNA copy numbers in harvested supernatants were quantified using a standard curve generated from in vitro transcripts via real-time RT-PCR. Viral titers ranged from 5.4 × 10^11^ to 5.8 × 10^11^ copies/mL. The isolates were subsequently processed for RNA extraction and complete genome sequencing. All procedures were conducted in a biosafety level 2 (BSL-2) facility, following standard biosafety protocols.

### 2.4. Complete Genome Sequencing

Viral RNA was extracted using the QIAamp Viral RNA Mini Kit (Qiagen, 52904, Hilden, Germany). Reverse transcription was performed with the SuperScript IV First-Strand Synthesis System (Invitrogen, Waltham, MA, USA) to produce cDNA. Overlapping PCR fragments covering the complete open reading frame (ORF, approximately 10. 3 kb) were amplified. PCR amplicons were subjected to paired- end sequencing (2 × 250 bp) on the Illumina MiSeq platform (Illumina, Inc., San Diego, CA, USA).

Raw sequence reads were assembled using both reference- guided and de novo methods in CLC Genomics Workbench (Qiagen), resulting in consensus sequences of roughly 10, 302 nucleotides. Complete ORFs from the three human-derived GV isolates were deposited in GenBank under accession numbers PQ442239 (K18P80), PQ442240 (K23P84), and PQ442241 (K23P88).

### 2.5. Phylogenetic and Mutation Analyses

For phylogenetic analysis, reference JEV sequences, including Korean isolates and vaccine strains, were retrieved from GenBank (NCBI, https://www.ncbi.nlm.nih.gov (accessed on 3 September 2024)) (Supplementary Data). Sequences were aligned using ClustalW in MEGA 11 software (https://www.megasoftware.net (accessed on 3 September 2024)), and phylogenetic trees were constructed using the maximum likelihood method with 1000 bootstrap replicates.

Nucleotide and amino acid sequence identities were determined using MEGA 11 and SimPlot v3.5.1 software. Amino acid substitutions were mapped across structural (E) and nonstructural proteins (NS1, NS2A, NS3, NS4B, and NS5a) to identify conserved mutations and clade- specific differences.

### 2.6. Structural Modeling and Visualization

The three-dimensional (3 D) structure of the Japanese encephalitis virus (JEV) envelope (E) protein was modeled based on the published crystal structure of the JEV SA 14-14-2 strain (PDB ID: 3 P 54). Amino acid substitutions identified in the Korean human-derived GV isolates (Q 52 E, S 156 T, and D 292 E) were mapped onto the template structure. Structural alignment, residue substitution, and visualization were performed using PyMOL version 3.1.6.1. (Schrödinger, LLC, New York, NY, USA). The positions of domains I–III were annotated per the established structural topology, and key substitutions were highlighted in stick representation. Figures were rendered at optimized ray-tracing resolution for publication-quality results.

## 3. Results

### 3.1. Phylogenetic Characterization and Clade Divergence of Human-Derived JEV GV Isolates in Korea

**Figure 1:**
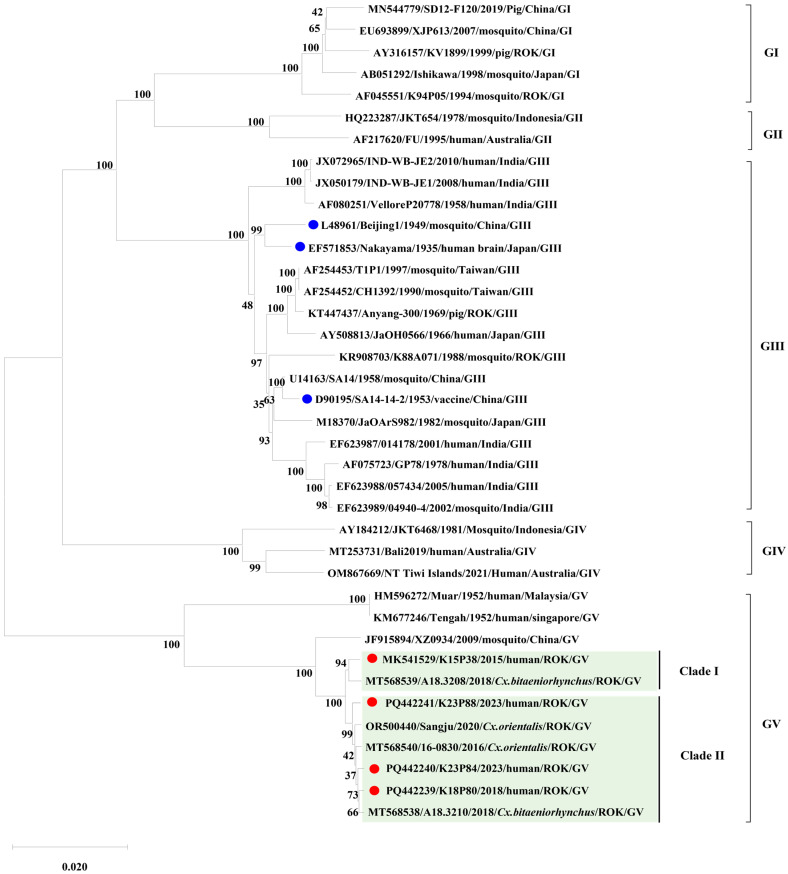
Phylogenetic analysis of Japanese encephalitis virus (JEV) isolates. Phylogenetic analysis was conducted using complete ORF sequences (10,302 nt) of Korean human-derived JEV GV isolates (K15P38, K18P80, K23P84, K23P88) together with representative reference strains of all five JEV genotypes (GI–GV), including vaccine strains (SA14-14-2, Nakayama, and Beijing-1). The tree was constructed using the maximum-likelihood method (1000 bootstrap replicates) in MEGA 11. Vaccine strains are indicated with blue dots, and Korean human-derived isolates are indicated with red dots to clearly distinguish the strain categories. Korean isolates clustered within genotype V (GV) and were clearly separated into two distinct clades: Clade I (K15P38) and Clade II (K18P80, K23P84, K23P88). Bootstrap support values ≥ 70% are indicated at the corresponding nodes. The scale bar represents nucleotide substitutions per site.

We performed phylogenetic analyses on the complete ORF sequences of three newly obtained human-derived isolates (K18P80, K23P84, K23P88), alongside the previously reported Korean human isolate K15P38 and representative JEV strains from genotypes GI–GV. The phylogenetic reconstruction showed that K18P80, K23P84, and K23P88 belong to the JEV GV lineage. However, these three isolates formed a distinct group separate from the 1952 Malaysian Muar strain and the 2009 Chinese XZ0934 strain. Notably, the earlier Korean human isolate K15P38 clustered independently from the new isolates, with K15P38 classified into Clade I, and K18P80, K23P84, and K23P88 forming Clade II (Figure 1).

This branching pattern aligned with the phylogenies of domestic mosquito-derived GV isolates with complete genomes. Interestingly, *Culex bitaeniorhynchus* mosquito specimens collected from the same pool in 2018 contained two GV isolates (A18.3208, Clade I; A18.3210, Clade II), which belonged to different clades. These findings indicate that JEV GV exhibits intra-genotypic diversification and circulates concurrently among humans and vectors in Korea.

### 3.2. Genetic Divergence of Human JEV Genotype V Isolates from Vaccine and Other Genotypes

**Figure 2:**
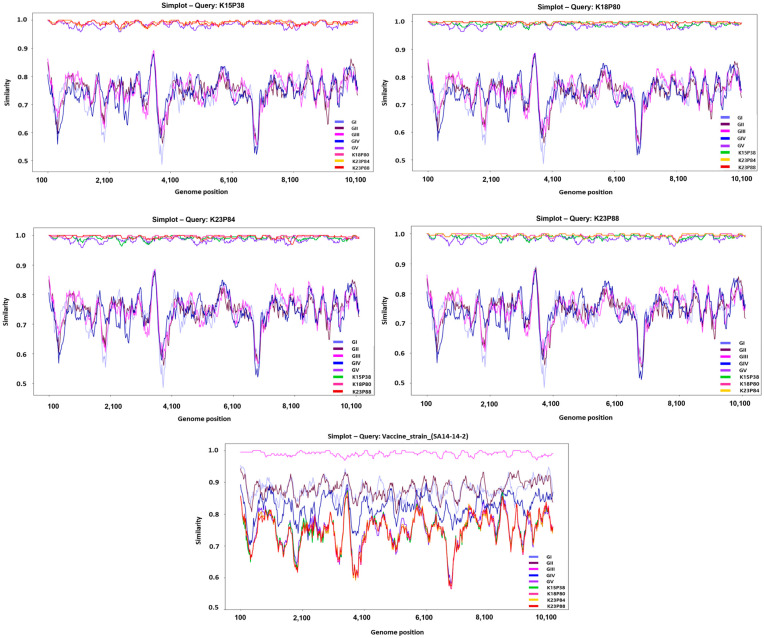
SimPlot analysis of domestic JEV GV isolates compared with reference strains. SimPlot-based nucleotide similarity analyses were performed using the four Korean human-derived JEV GV isolates, K15P38, K18P80, K23P84, and K23P88, as query sequences. Each panel shows the nucleotide similarity (%) between the query sequence and representative reference strains of the five JEV genotypes (GI–GV), calculated across the complete ORF (10,302 nt) using a sliding-window approach (window size: 200 nt; step size: 20 nt). Genotype-specific similarity profiles are color-coded (right legend), and the gray dashed line marks the 80% similarity threshold. All Korean isolates consistently exhibited the highest similarity to the GV reference strain, clearly differentiating them from GI–GIV and vaccine strains.

Across the three newly obtained isolates, complete nucleotide sequence identity with other JEV genotypes (GI–GV) averaged 79% (range 78.8–79.2%), whereas amino acid identity averaged 91.3% (range 91–92%). Compared to vaccine strains (SA14-14-2, Nakayama, Beijing-1), nucleotide and amino acid identities were similarly low at 79% and 91.1%, respectively. Genome similarity analyses revealed regions of markedly reduced nucleotide identity at approximately 4000–5000 nt and near 7000 nt, corresponding to nonstructural protein coding regions. This finding highlights the substantial genetic dynamics in nonstructural proteins compared to structural proteins, indicating divergence from existing vaccine strains (Figure 2).

### 3.3. Distinct Amino Acid Substitutions and Molecular Signatures in Human-Derived JEV Genotype V Isolates

**Figure 3:**
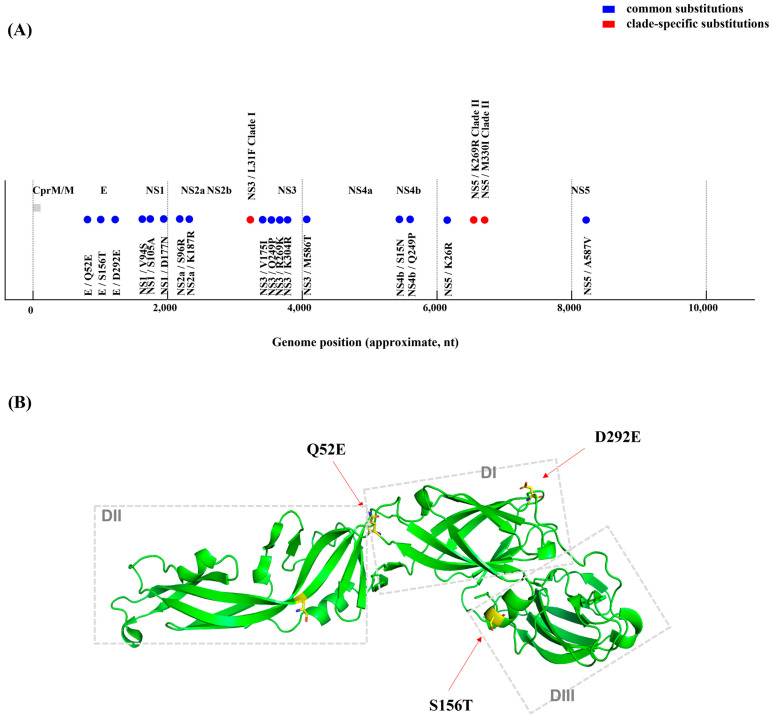
Polyprotein schematic and key amino acid substitutions. (**A**) Genomic distribution of amino acid substitutions identified in Korean human-derived JEV genotype V isolates. Schematic representation of the complete JEV polyprotein (~3430 amino acids, ~10,302 nucleotides) showing the positions of structural and nonstructural proteins (C, prM/M, E, NS1–NS5). Blue circles indicate amino acid substitutions commonly identified in all human-derived GV isolates from Korea, including E (Q52E, S156T, D292E), NS1 (V94S, S105A, D177N), NS2A (S96R, K187R), NS3 (V175I, Q249P, R269K, K304R, M586T), NS4B (S15N, R84K), and NS5a (K26R, A587V). Red circles represent clade-specific substitutions distinguishing Clade I and Clade II isolates, namely NS3 (F31L) and NS5a (K269R, M330I). (**B**) Predicted three-dimensional structure of the JEV E protein highlighting key amino acid substitutions. The tertiary structure of JEV E protein was visualized using PyMOL version 4.6. The structure illustrates the overall *β*-barrel architecture of domains I–III, with the three Korean human-derived GV signature substitutions (Q52E, S156T, and D292E) highlighted in yellow and represented as stick models. These residues are located in the domain I hinge region, adjacent to the N154 glycosylation site, and at the domain II/III interface, respectively; these regions are structurally linked to antigenic epitopes and neutralizing antibody binding sites. These amino acid changes were mapped across the polyprotein backbone and correspond to functionally relevant domains involved in viral replication, immune evasion, and antigenic recognition. Identical clade-specific markers were also observed in mosquito-derived GV isolates, indicating a molecular link between human and vector lineages.

Comparative amino acid sequence analyses were conducted on all four Korean human-derived GV isolates (K15P38, K18P80, K23P84, and K23P88) against foreign human-derived GV strains (Muar, Tengah) and vaccine strains. We identified amino acid substitutions unique to the Korean isolates. In the E protein, consistent substitutions, Q52E, S156T, and D292E, were observed in all Korean isolates. These substitutions are located in the domain I hinge region, adjacent to the N154 glycosylation site, and at the domain II/III interface, all of which are structurally linked to antigenic epitopes and neutralizing antibody binding sites (Table 1 and Table S2). Multiple substitutions were also detected within nonstructural proteins: NS1 (V94S, S105A, D177N), NS2A (S96R, K187R), NS3 (V175I, Q249P, R269K, K304R, M586T), NS4B (S15N, R84K), and NS5A (K26R, A587V). Interestingly, these recurring substitutions have also been observed in mosquito-derived GV sequences as documented in previous reports (Figure 3, Table 1 and Table S2).

### 3.4. Molecular and Phylogenetic Continuity Between Human- and Mosquito-Derived JEV Genotype V Isolates

Phylogenetic analyses showed that both human- and mosquito-derived GV isolates in Korea are divided into two distinct groups (Clade I and Clade II). These clades are differentiated by specific amino acid substitutions in nonstructural proteins NS3 and NS5. Clade I is characterized by an NS3 L31F substitution, whereas Clade II features NS5 K269R and M330I substitutions (Table 1 and Table S2). This pattern of clade differentiation was confirmed in GV isolates obtained from *Culex bitaeniorhynchus* mosquitoes collected at Camp Humphreys in 2018. Specifically, A18.3208 (Clade I) had the same substitution profiles as K15P38, whereas A18.3210 (Clade II) matched the substitution patterns of K18P80, K23P84, and K23P88. These findings strongly support the molecular link between domestic human-derived GV isolates and vector-derived GV strains.

## 4. Discussion

This study analyzed complete genomes of human-derived JEV GV isolates from Korea to elucidate their molecular phylogenetic characteristics, providing insights into the present distribution and evolutionary dynamics of GV in the country. GV was first identified in Malaysia in 1952 but remained undetected for decades until it re-emerged in China in 2009 and later in Korea during the 2010s, drawing attention as a ‘re-emerging virus’ [9,10]. A previous Korean study reported that mosquito-derived GV strains fell into two distinct groups (Clades I and II), indicating intra-lineage diversification within local vectors [14]. In the present study, we also examined three human-derived isolates. We confirmed that they segregated into the same two clades, clearly demonstrating genetic continuity between human and mosquito populations. Notably, both clades were detected within a single mosquito species, and clade-specific amino acid substitutions were identified, providing molecular evidence of ongoing diversification and the human–vector linkage of JEV GV in Korea. Three amino acid substitutions in the E protein (Q52E, S156T, D292E) were consistently found in all Korean human-derived GV isolates. These substitutions may have important implications for antigenicity and immune evasion. Q52E is located in the hinge of domain I, which may alter the relative domain orientations and the overall protein flexibility, thereby affecting antibody accessibility and fusion function [18,19]. S156T lies adjacent to the N154 glycosylation site, glycosylation at N154 is crucial for particle stability, host receptor binding, and immune evasion in JEV and other *Orthoflaviviruses*, indicating that S156T could influence glycosylation efficiency, affecting virus secretion, antibody recognition, and neutralization [20]. D292E lies in the DI–DIII linker near a conserved basic residue cluster, where it may alter local electrostatics and flexibility, potentially affecting receptor and antibody interactions and contributing to genotype-specific differences in immune recognition [21]. Overall, these mutations may be signature changes maintained by host immune pressure. The high level of polymorphism observed in NS regions also has important evolutionary implications. Several studies on *Orthoflaviviruses* indicate that NS protein differences directly affect replication efficiency, host range, and antiviral resistance [22,23]. As NS proteins are involved in viral RNA replication, protein processing, and immune evasion, the extensive variability identified in the present study suggests that Korean GV strains are adapting to local hosts and ecological pressures, forming independent evolutionary trajectories rather than representing simple re-emergence events. All isolates in the present study originated from unvaccinated individuals, which is important for interpretation. The E protein substitutions and NS diversity observed likely reflect intrinsic GV evolution, independent of vaccine-driven selection. The consistently low genetic similarity to GIII-based vaccine strains indicates long-term divergence and sustained circulation, rather than simply vaccine pressure. This indicated that cross-protective immunity might be suboptimal in both vaccinated and unvaccinated populations, introducing a new variable into GV transmission dynamics and control strategies. Diagnostically, the fact that all viral isolates were detected exclusively in CSF, not serum, aligns with previous reports of brief viremia during early JEV infection [24,25]. This highlights the need for CSF-based diagnostics and emphasizes appropriate sample collection for accurate JE diagnosis, informing improvements to national surveillance and clinical protocols. This study has several limitations. The clade-specific amino acid patterns were derived from only four human isolates, which may limit the generalizability of the findings. The viral stocks used for sequencing underwent only one or two passages, and their high concordance with mosquito-derived GV sequences suggests that passage-related mutations, if present, were minimal. Although functional assays were not performed, future studies using reverse genetics, structural modeling, or neutralization assays will be important to determine whether the identified substitutions influence viral antigenicity, receptor interactions, or immune modulation. In summary, the identification of consistent E protein substitutions and NS region polymorphisms in human GV isolates, and their concordance with domestic mosquito-derived sequences, demonstrates that GV in Korea is undergoing persistent human–vector transmission with locally adapted evolutionary patterns, rather than functioning solely as a reintroduced genotype.

## 5. Conclusions

Through comprehensive genome analysis of four human-derived JEV GV isolates from Korea, we identified two distinct co-circulating clades (Clades I and II) within the domestic GV population. These isolates showed clear genetic and antigenic differences from genotype III (GIII) vaccine strains, indicating possible limitations in cross-protective effectiveness. Additionally, identical molecular phylogenetic features between human- and mosquito-derived isolates suggest ongoing local circulation between human and vector populations. This study emphasizes the importance of CSF-based diagnosis and provides essential baseline data for the advancement of next-generation JEV vaccine and improved surveillance strategies.

## Figures and Tables

**Table 1:** Characteristic amino acid substitutions in human-derived genotype V Japanese encephalitis virus isolates identified in Korea. Amino acid substitutions identified in the structural (E) and nonstructural (NS1–NS5) proteins of human-derived Japanese encephalitis virus (JEV) genotype V isolates from Korea are summarized. Substitutions commonly observed among all isolates are presented under the “Common substitutions” column, whereas clade-specific variations are distinguished for Clade I (K15P38) and Clade II (K18P80, K23P84, K23P88). The functional relevance of each substitution was inferred based on protein domain analyses and computational predictions, highlighting the potential roles of these residues in viral replication, antigenicity, glycosylation, and immune evasion.

Protein	Common Substitutions (All Korean GV Isolates)	Clade I (K15P38) Specific	Clade II (K18P80, K23P84, K23P88) Specific	Functional Relevance (Predicted)
E (envelope)	Q52E, S156T, D292E	–	–	Antigenic sites; fusion domain; glycosylation-associated
NS1	V94S, S105A, D177N	–	–	Replication complex formation; vascular permeability
NS2a	S96R, K187R	–	–	Membrane association; immune evasion
NS3	V175I, Q249P, R269K, K304R, M586T	F31	L31	Protease/helicase activity; RNA replication
NS4b	S15N, R84K	–	–	Membrane interaction; innate immunity modulation
NS5a	K26R, A587V	–	K269R, M330I	RNA polymerase activity; replication fidelity

## Data Availability

The data that support the findings of this study will be available in GenBank at https://www.ncbi.nlm.nih.gov/genbank/ (accessed on 2 December 2025) under the accession numbers PQ442239–PQ442241 following an embargo period from the date of publication due to commercial restrictions. Supporting alignment and phylogenetic tree files will be deposited in an open-access repository upon acceptance.

## Supplementary Materials

The following supporting information can be downloaded at: https://www.mdpi.com/article/10.3390/pathogens14121279/s1, Table S1: Metadata of Japanese encephalitis virus (JEV) sequences analyzed in this study; Table S2: Characteristic amino acid substitutions identified in Korean human-derived JEV GV isolates and their putative functional implications.

## Abbreviations

The following abbreviations are used in this manuscript:

JEV	Japanese encephalitis virus
CSF	Cerebrospinal fluid
GV	Genotype V
