# Supplementary material for: Complete Genome Sequences of Human Japanese Encephalitis Virus Genotype V Isolates in Korea Reveal Genotype-Specific Amino Acid Signatures
Source: Pathogens. 2025 Dec 12;14(12):1279. doi: 10.3390/pathogens14121279 (PMC12735970; doi:10.3390/pathogens14121279)
Supplement: Supplementary file 1 [file pathogens-14-01279-s001.zip › pathogens-4011975-supplementary.pdf]

**Supplementary Data**

**Table S1. Metadata of Japanese encephalitis virus (JEV) sequences analyzed in this study.** A total of 39 sequences were obtained from the NCBI GenBank database, including accession number, strain name, year of isolation, host, country, and genotype. These sequences were used for phylogenetic and comparative genomic analyses.

| No. | Accession no. | Strain | Year | Host | Country | Genotype |
|---|---|---|---|---|---|---|
| 1 | AF045551 | K94P05 | 1994 | *Culex tritaeniorhynchus* | ROK | GI |
| 2 | AB051292 | Ishikawa | 1998 | mosquito (unspecified) | Japan | GI |
| 3 | AY316157 | KV1899 | 1999 | pig | ROK | GI |
| 4 | EU693899 | XJP613 | 2007 | mosquito (unspecified) | China | GI |
| 5 | MN544779 | SD12-F120 | 2019 | pig | China | GI |
| 6 | HQ223287 | JKT654 | 1978 | mosquito (unspecified) | Indonesia | GII |
| 7 | AF217620 | FU strain | 1995 | human | Australia | GII |
| 8 | EF571853 | Nakayama | 1935 | vaccine (human brain) | Japan | GIII |
| 9 | L48961 | Beijing1 | 1949 | Vaccine (mosquito) | China | GIII |
| 10 | D90195 | SA14-14-2 | 1953 | vaccine | China | GIII |
| 11 | AF080251 | Vellore P20778 | 1958 | human | India | GIII |
| 12 | U14163 | SA14 | 1958 | mosquito (unspecified) | China | GIII |
| 13 | AY508813 | JaOH0566 | 1966 | human | Japan | GIII |
| 14 | KT447437 | Anyang-300 | 1969 | pig | ROK | GIII |
| 15 | AF075723 | GP78 | 1978 | human | India | GIII |
| 16 | M18370 | JaOArS982 | 1982 | mosquito (unspecified) | Japan | GIII |
| 17 | KR908703 | K88A071 | 1988 | mosquito (unspecified) | ROK | GIII |
| 18 | AF254452 | CH1392 | 1990 | mosquito (unspecified) | Taiwan | GIII |
| 19 | AF254453 | T1P1 | 1997 | mosquito (unspecified) | Taiwan | GIII |

| | | | | | | |
|---|---|---|---|---|---|---|
| 20 | EF623987 | 14178 | 2001 | human | India | GIII |
| 21 | EF623989 | 04940-4 | 2002 | mosquito (unspecified) | India | GIII |
| 22 | EF623988 | 57434 | 2005 | human | India | GIII |
| 23 | JX050179 | IND-WB-JE1 | 2008 | human | India | GIII |
| 24 | JX072965 | IND-WB-JE2 | 2010 | human | India | GIII |
| 25 | AY184212 | JKT6468 | 1981 | mosquito (unspecified) | Indonesia | GIV |
| 26 | MT253731 | Bali2019 | 2019 | human | Australia | GIV |
| 27 | OM867669 | NT Tiwi Islands | 2021 | human | Australia | GIV |
| 28 | HM596272 | Muar | 1952 | human | Malaysia | GV |
| 29 | KM677246 | Tengah | 1952 | human | singapore | GV |
| 30 | JF915894 | XZ0934 | 2009 | mosquito | China | GV |
| 31 | JN587258 | 10-1827 | 2010 | *Culex bitaeniorhynchus* | ROK | GV |
| 32 | KJ420589 | K12HC959 | 2012 | *Culex orientalis* | ROK | GV |
| 33 | KJ420590 | K12AS1148 | 2012 | *Culex pipiens* | ROK | GV |
| 34 | KM496503 | K13GB57 | 2013 | *Culex tritaeniorhynchus* | ROK | GV |
| 35 | MK541529 | K15P38 | 2015 | human | ROK | GV |
| 36 | MT568540 | 16-0830 | 2016 | mosquito (unspecified) | ROK | GV |
| 37 | MT568538 | A18.3210 | 2018 | *Culex bitaeniorhynchus* | ROK | GV |
| 38 | MT568539 | A18.3208 | 2018 | *Culex bitaeniorhynchus* | ROK | GV |
| 39 | PQ442239 | K18P80 | 2018 | human | ROK | GV |
| 40 | OR500440 | Sangju | 2020 | *Culex orientalis* | ROK | GV |
| 41 | PQ442240 | K23P84 | 2023 | human | ROK | GV |
| 42 | PQ442241 | K23P88 | 2023 | human | ROK | GV |

**Table S2. Characteristic amino acid substitutions identified in Korean human-derived JEV GV isolates and their putative functional implications**

Amino acid substitutions identified in the E and NS proteins of Korean human-derived JEV GV isolates are summarized. Residues are listed with their genomic position and substitution, along with functional domains previously implicated in viral replication, antigenicity, or immune modulation. References indicate prior studies describing their structural or functional significance.

| Accession | Strain | Year | Host | E | | | NS1 | | | NS2a | | NS3 | | | | | | NS4b | | NS5a | | | |
|---|---|---|---|---|---|---|---|---|---|---|---|---|---|---|---|---|---|---|---|---|---|---|---|
| | | | | 52 | 156 | 292 | 94 | 105 | 177 | 96 | 187 | 31 | 175 | 249 | 269 | 304 | 586 | 15 | 84 | 26 | 269 | 330 | 587 |
| D90195 | SA14-14-2 | 1953 | vaccine | Q | S | D | V | S | D | S | K | L | V | Q | R | K | M | S | R | K | K | M | A |
| EF571853 | Nakayama | 1935 | human brain | Q | S | D | V | S | D | S | K | L | V | Q | R | K | M | S | R | K | K | M | A |
| L48961 | Beijing1 | 1949 | mosquito | Q | S | D | V | S | D | S | K | L | V | Q | R | K | M | S | R | K | K | M | A |
| HM596272 | Muar | 1952 | human | Q | S | D | V | S | D | S | K | L | V | Q | R | K | M | S | R | K | K | M | A |
| KM677246 | Tengah | 1952 | human | Q | S | D | V | S | D | S | K | L | V | Q | R | K | M | S | R | K | K | M | A |
| JF915894 | XZ0934 | 2009 | *Cx.tritaeniorhynchus* | E | T | E | S | T | N | R | R | L | I | Q | R | R | T | N | K | R | K | I | V |
| JN587258 | 10-1827 | 2010 | *Cx.bitaeniorhynchus* | E | T | E | | | | | | | | | | | | | | | | | |
| KJ420589 | K12HC959 | 2012 | *Cx.orientalis* | E | T | E | | | | | | | | | | | | | | | | | |
| KJ420590 | K12AS1148 | 2012 | *Cx.pipiens* | E | T | E | | | | | | | | | | | | | | | | | |
| KM496503 | K13GB57 | 2013 | *Cx.tritaeniorhynchus* | E | T | E | | | | | | | | | | | | | | | | | |
| MT568540 | 16-0830 | 2016 | *Cx.orientalis* | E | T | E | S | A | N | R | R | L | I | P | K | R | T | N | K | R | K | I | V |

| | | | | | | | | | | | | | | | | | | | | | | | |
|---|---|---|---|---|---|---|---|---|---|---|---|---|---|---|---|---|---|---|---|---|---|---|---|
| OR500440 | Sangju | 2020 | *Cx. orientalis* | E | T | E | S | A | N | R | R | L | I | Q | K | R | T | N | K | R | K | I | V |
| MK541529 | K15P38 | 2015 | human | E | T | E | S | A | N | R | R | F | I | P | K | R | T | N | K | R | K | M | V |
| MT568539 | A18.3208 | 2018 | *Cx.bitaeniorhynchus* | E | T | E | S | A | N | R | R | F | I | P | K | R | T | N | K | R | K | M | V |
| MT568538 | A18.3210 | 2018 | *Cx.bitaeniorhynchus* | E | T | E | S | A | N | R | R | L | I | P | K | R | T | N | K | R | R | I | V |
| PQ442239 | K18P80 | 2018 | human | E | T | E | S | A | N | R | R | L | I | P | K | R | T | N | K | R | R | I | V |
| PQ442240 | K23P84 | 2023 | human | E | T | E | S | A | N | R | R | L | I | P | K | R | T | N | K | R | R | I | V |
| PQ442241 | K23P88 | 2023 | human | E | T | E | S | A | N | R | R | L | I | P | K | R | T | N | K | R | R | I | V |
